# Underwater Sensor Nodes and Networks

**DOI:** 10.3390/s130911782

**Published:** 2013-09-05

**Authors:** Jaime Lloret

**Affiliations:** Instituto de Investigación para la Gestión Integrada de Zonas Costeras (IGIC), Universidad Politécnica de Valencia, Camino Vera s/n, Valencia 46022, Spain; E-Mail: jlloret@dcom.upv.es; Tel.: +34-609-549-043; Fax: +34-962-849-313

**Keywords:** underwater sensor nodes, underwater sensor networks, UWSNs, underwater sensor communications

## Abstract

Sensor technology has matured enough to be used in any type of environment. The appearance of new physical sensors has increased the range of environmental parameters for gathering data. Because of the huge amount of unexploited resources in the ocean environment, there is a need of new research in the field of sensors and sensor networks. This special issue is focused on collecting recent advances on underwater sensors and underwater sensor networks in order to measure, monitor, surveillance of and control of underwater environments. On the one hand, from the sensor node perspective, we will see works related with the deployment of physical sensors, development of sensor nodes and transceivers for sensor nodes, sensor measurement analysis and several issues such as layer 1 and 2 protocols for underwater communication and sensor localization and positioning systems. On the other hand, from the sensor network perspective, we will see several architectures and protocols for underwater environments and analysis concerning sensor network measurements. Both sides will provide us a complete view of last scientific advances in this research field.

## 1. Introduction

More than the 70% of the Earth's surface is covered with water (almost all of that are seas and oceans). New technologies have brought us new ways to monitor and sense aquatic environments. Marine surveillance, river and sea pollution detection and monitoring, and oceanographic data collection are needed to explore, protect, and commercial exploit the aquatic environment. Many potential applications exist in this environment such as fish and mussels grow observation [1,2], deep-sea archaeology, seismic and volcanic prediction, oil monitoring and so on.

Sensor technology has matured enough to be used in any type of environment. As time goes on, the market offers smaller sensor devices, with lower power consumption and higher processing capacity. Moreover, the appearance of new physical sensors has increased the range of aquatic parameters for gathering data. Because of the huge amount of unexploited resources in the ocean environment, there is a need of new research lines in the field of sensors and sensor networks [3]. Furthermore, many sensor and sensor networks features must be taken into account in this type of environment: the need of large number of nodes, limited energy of the nodes, short-distance radio communications, large propagation latency, low bandwidth capacity and high error rate. All these constraints make underwater sensors and underwater sensor networks a special case of the regular ones.

This special issue has tried to collect the recent advances on underwater sensors and underwater sensor networks. It has been mainly focused on measuring, monitoring, surveillance and controlling underwater environments. The topics of interest in the call for papers are shown in Table 1.

**Figure 1.**
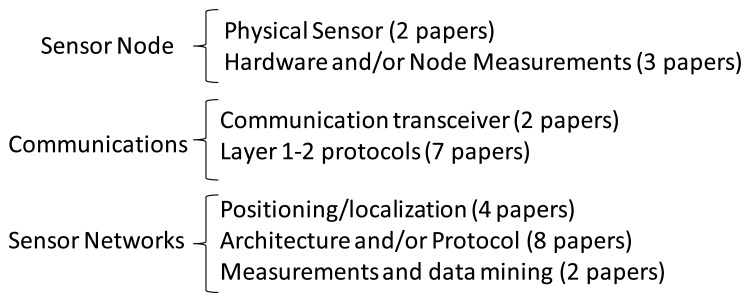
Special Issue Papers classification.

This Special Issue has finally included 28 papers, which have been classified as shown in Figure 1. Five papers focused on the underwater sensor node (with the topics physical sensor, hardware and node measurements) have been accepted, nine papers focused on underwater communications (with the topics communication transceiver and layer 1–2 protocols) have been accepted, and 14 papers on sensor networks (with the topics position/location, architecture and protocol, and sensor network measurements) have been accepted. It has been split in this manner because the papers included in the underwater communication item have not tackled the problem from the network perspective, but on a single communication between two sensor nodes.

**Figure 2.**
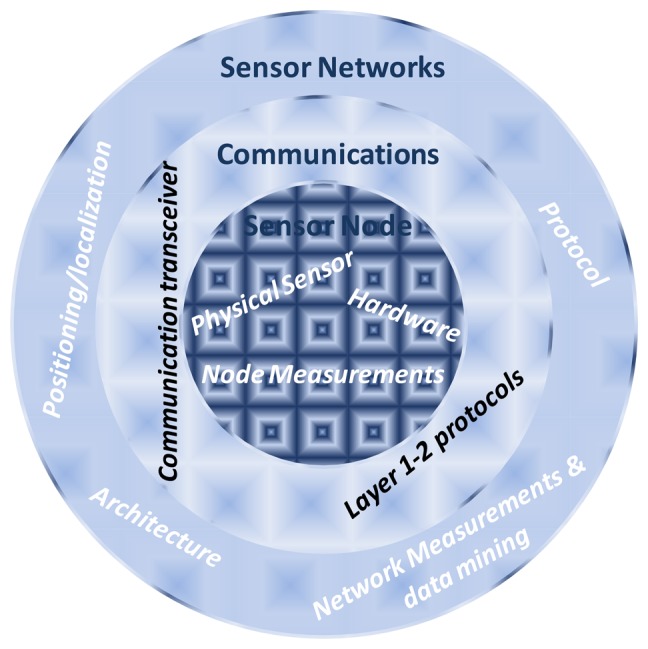
Classification of the topics into levels.

To provide a better view of this classification when studying every item, we also present Figure 2, which splits this classification into levels. It shows that in order to develop and deploy an underwater sensor network, we should take into account the positioning and location of the sensor nodes, its architecture and protocol, but we should also take into account the lower level: the underwater communications, where we should take into account the communication transceiver and the layer 1–2 protocol. Moreover, this level has no sense without the lower level: sensor node, where we should take into account the physical sensor and the node hardware.

The structure of the paper is as follows: Section 2 presents the papers published in this Special Issue in the topic Sensor Nodes. The papers included in underwater communications topics are described in Section 3. Section 4 discusses the papers included in the underwater sensor networks topic. Finally Section 5 draws the conclusions of this Special Issue.

## 2. Underwater Sensor Nodes

This section shows the papers accepted in the Special Issue focused on underwater sensor node topics.

In [4], Martinez *et al.* design, build, and test a portable, watertight, and user-friendly an autonomous underwater sound recording device (USR) that monitors the underwater sound and pressure waves generated by anthropogenic activities, such as underwater blasting and pile driving. It uses two hydrophones or other dynamic pressure sensors which allow approximately up to 1 h and 55 min of data collection from both sensors simultaneously. The gain can be independently set for each sensor. They proposed two versions. The first one is a submersible model deployable to a maximum depth of 300 m and the second one is a watertight but not fully submersible model. The submersible version is appropriate for collecting long-duration measurements at depths that would require very long hydrophone cables or extension cables, while the non-submersible version is better for short duration underwater monitoring cases. Their test demonstrates that the device operates as designed and performs as well as larger commercially available data acquisition systems. Moreover, they present a case study with a prototype measuring blast pressures while investigating the effect of underwater rock blasting events while simultaneously exposing juvenile Chinook salmon and rainbow trout held in cages to the same sound and pressure waves. Device withstood operation in harsh environments, thus it is a valuable tool for collecting field measurements.

Ardid *et al.* present in [5] the development of acoustic transmitters an underwater neutrino telescope (KM3NeT). Firstly, they deployed an acoustic transceiver as part of the acoustic positioning system in order to monitor the position of the optical sensors which move due to sea currents. The transceiver has reduced cost, low power consumption, high pressure withstanding (up to 500 bars), high intensity for emission, low intrinsic noise, arbitrary signals for emission and the capacity of acquiring and processing received signals. Secondly, they deployed a compact acoustic transmitter array for the calibration of acoustic neutrino detection system, which is able to mimic the signature of ultra-high-energy neutrino interaction in emission directivity and signal shape. They present the successful results of the tests and measurements performed with FFR-SX30 hydrophones and a custom sound emission board. Due to the versatility of the transceiver system, this prototype could be implemented to carry out several calibration tasks related to acoustic emission in underwater neutrino telescopes. Moreover, it can be used in other applications, such as marine positioning systems or integrated in different Earth-Sea Observatories, where the localization of the sensors is an issue, especially when very directive beams are required and/or signal processing techniques are needed.

The paper authored by Baronti *et al.* [6], presents an electronic meter to measure surface seawater density based on the buoyancy force measurement carried out using a magnetostrictive linear displacement sensor. It uses an Archimedes' law (measuring the difference in displacements of a surface level probe and a weighted float, the density of the water can be estimated). They describe the operating principles of the meter, the design of its mechanical and electronic parts and its features. The displacements are simultaneously measured using magnetostrictive sensors. The hardware used is a custom electronic board, which provides a wireless connection and power supply. The wireless module and the firmware of the microcontroller can be changed, which means that the node can be easily used in underwater or above surface wireless networks. Presented laboratory and at-sea tests show the performance of a wireless density meter node using Bluetooth. The authors are satisfied with the accuracy of the system compared to reference hydrometer readings. It had good response even in the noisy environment caused by sea waves.

The Ocean Bottom Seismometer (OBS) presented by Manuel *et al.* in [7] has been designed to be used in long duration seismic surveys. It has low power consumption and it is able to store large data with high resolution and Signal-to-Noise Ratio (SNR). One of its key points is the noise level of the acquisition system. Throughout the deployment of the marine seismometer prototype, calibration standards (such as “IEEE Std-1057 Standard for Digitizing Waveform Recorders” and “IEEE Std-1241 Standard for Terminology and Test Methods for Analog-to-Digital Converters”) have been used in order to let them know the uncertainties in the measurements Moreover, the geophone has been designed to obtain maximum sensitivity. In a seismic survey, a series of OBSs are placed on the seabed of the area under study, where they record either natural seismic activity or acoustic signals generated by compressed air-guns on the ocean surface, and the motion of the ocean floor in order to study offshore seismicity and to explore the Earth's crust. The resulting data sets are subsequently used to model both the earthquake locations and the crustal structure. The deployed OBS can be transported and manipulated in a small ship, and an oceanographic vessel is not needed. Moreover, it can be used in civil engineering, submarine surveying, and mine prospecting.

The effects on ecological productivity and biogeochemical cycling can be evaluated by determining how land-use changes affect dissolved organic carbon (DOC) concentrations and bioavailability within aquatic ecosystems. In [8], Jollymore *et al.* present a case study of a submersible UV-Vis absorbance spectrophotometer to determine stream organic carbon dynamics within a headwater catchment. The experiment was placed near Campbell River (on the eastern side of Vancouver Island, British Columbia, Canada). Field measurements were made before and after forest harvest. The system has high temporal resolution compared to traditional grab sampling and laboratory measurements, which allows resolving variations at small time scales and the observation of concentration changes over longer time periods, and thus demonstrating DOC concentration dynamics over the pre and post harvest periods. One of its main advantages is the ability to remotely monitor the system through a wireless connection. The system is able to react when environmental or power disturbances cause system interruption and data loss.

## 3. Underwater Communications

Won and Park show the design and Implementation of an Omni-Directional Underwater Acoustic Micro-Modem Based on a Low-Power Micro-Controller Unit in [9]. The work provides the development of the cylindrically shaped micro-modem guaranteeing an omni-directional beam pattern in three dimensions. The system uses an ARM Cortex-M3 is embedded in the micro-modem to provide fast digital signal processing and to support flexible interfaces with other peripherals, and a spherical transducer for the implementation. The size of the developed cylindrical modem is only 70 mm in diameter and 35 mm in height. The authors also implemented in software and hardware the physical layer frame format and symbol structure for efficient packet-based underwater communication systems. Experiments were executed in a water tank, a pond and a river, obtaining expected results by analysis. Their experiment in the river showed that the modem was able to send data wirelessly up to 70 m with a data rate of 0.2 kbps while consuming a power of 4.5 watts. But it can be increased using a higher supply voltage or a transducer having lower resonant frequency. The modem can be used for various UWSN applications requiring acoustic communication taking profit of its features (low-power consumption, micro size, low cost, omni-directivity).

The paper in [10], authored by Sanchez *et al.*, describes the physical layer of a new low-cost acoustic modem called ITACA. Its architecture combines a typical microcontroller-based core with energy-efficient mechanisms. One of the main features of the modem is the implementation of an ultra-low power asynchronous wake-up system for underwater acoustic transmission that is based on a low-cost off-the-shelf RFID peripheral integrated circuit. This allows a reduced power dissipation in stand-by mode and very low power values during reception and transmission. The modem uses coherent-FSK modulation. It also incorporates clear channel assessment (CCA) detection using both carrier frequency detection and the RSSI level to support CSMA-based medium access control (MAC) layer protocols and an efficient development of upper communication layers by supporting additional RTC (Real Time Clock) asynchronous wake-up and clear channel assessment (CCA). This asynchronous wake-up design has been tested for non-detections and false positives with excellent results.

Park, and Shin propose in [11] the Selective Multiple Acknowledgement (SMA) method, based on Multiple Acknowledgement (MA), to efficiently reduce the amount of data transmission in an underwater environment. The transmission technique collectively transmits Acknowledgement (Ack) or Negative Acknowledgement (Nack) in order to pro information regarding the existence (or non-existence) of transmission errors. Ack and Nack are included in the Beacon frame. There is a Cluster Head (CH), which supervises scheduling and data collection, collects data via Uplink transmission within a unit cluster. Authors redesigned the transmission frame structure and took into consideration underwater transmission characteristics, which let it handle the same amount of data in a much more compact frame structure without any appreciable loss of reliability. Its method significantly reduces the number of transmissions and the frame length (which increases according to the number of participating network nodes containing Ack). They analyzed and compared in terms of performance their proposal with the conventional Automatic Repeat-reQuest (ARQ), Block Acknowledgement (BA), block response, and MA methods, obtaining better results. The proposed SMA present the smallest frame size and the lowest number of transmission with various variables. As the number of transmission targets increases, the performance of the proposed technique shows better performance, thus the efficiency of the underwater sensor network, which forms a large cluster and mostly contains uplink data is improved by the proposed method.

Kim and Park propose in [12] a novel multiple query result merging scheme for underwater sensor networks. It is based on a relational database model and is adjusted to the practical restrictions affecting underwater communication environments. It is an energy-efficient technique for providing data on-demand in an underwater sensor network to multiple-users simultaneously. They also proposed a message payload format capable of maintaining robustness in the face of unexpected communication failures. Network simulations prove that the query management scheme can efficiently reduce redundant message transmissions and the entailed energy waste, and enhance network lifetime. It becomes more efficient with a greater number of generated queries and a relatively smaller period range.

In [13], Llor and Malumbres have studied how acoustic propagation models impact the performance of high level communication protocols. First, they analyzed the evolution of underwater acoustic prediction models. Second, they analyzed the performance behavior of different MAC protocols and also checked how the scenario environmental changes impact on their network performance in terms of throughput, delay and collisions. They tested different high layer network protocols (MAC and routing protocols) from a simple to more detailed and accurate models in order to determine the influence of environmental parameters on the obtained results. They concluded that higher-level protocols are sensitive to both, physical layer parameters related to the network scenario (like ocean surface activity, scenario location, bathymetry or floor sediment composition) and the acoustic propagation model (in terms of accuracy and low-complexity). In the paper, they show the importance of defining an accurate and low complexity propagation model, and the sensibility of higher layer protocols to the time-varying scenario environmental conditions. They found that accurate and low-complexity propagation models are required for network simulation in order to obtain reliable results attained to the specific scenario and environmental parameters. This information should be taken into account when designing UWSN architectures for real network scenarios.

Zhang *et al.* show in [14] how to remove intersymbol interference (ISI), and evaluate the performance of the implementation of three different receiver structures using real data collected in a high-frequency (10–14 kHz) field experiment. The three structures were multichannel decision feedback equalizer (McDFE), passive time reversal receiver (passive-phase conjugation (PPC) with a single channel DFE), also called PPC-DCE, and the joint PPC with multichannel DFE (PPC-McDFE). In order to test the scenario, the information of the channel characteristics for PPC processing was obtained by using the two shaping pulses: a linear frequency modulation pulse (LFM) chirp as a channel probe, and a root-raised-cosine pulse (RRC). They observed that the difference between LFM and RRC is minimal. The three structures were evaluated by off-line data processing. In the assessment, they found that it is advantageous to obtain spatial gain using the adaptive multichannel combining scheme and the matching pursuit (MP) algorithm improves the performance of communications using PPC processing. They observed that PPC-DFE achieved the worst performance in the assessment, and the performance of PPC-McDFE approximated that of McDFE. Time-variant reverberations resulted in unpredictable spatial coherence, which may impact on the performance of PPC-DFE. Therefore, it is preferable to use PPC-McDFE instead of PPC-DFE in a channel of long time spread. Moreover, they observed that PPC-McDFE is less sensitive to the channel variations than PPC-DFE.

In [15], Ramezani and Leus show how the underwater localization case can be converted to the traditional range-based terrestrial localization case when the depth information of the nodes is known *a priori*, because underwater medium is assumed to be composed of multiple isogradient sound speed profile (SSP) layers where in each layer the sound speed is linearly related to the depth. They used a ray-tracing approach for high-frequency signal transmission to model the propagation and a set of polynomial root finding equations to distinguish among different possible transmission paths between the nodes, and determine the fastest one. Each sensor node is able to measure its depth and can exchange this information with other nodes. Then, they relate the pair-wise time of flight (ToF) values with nodes positions in order to propose a novel ranging algorithm for an underwater medium. The proposed ranging algorithm considers reflections from the seabed and sea surface and selects the fastest transmission path based on the measured ToF and the nodes' depth measurements. The algorithm estimates iteratively the range of a target by minimizing the difference between the measured ToF and the constructed ToF estimated from the known map. The authors evaluate the performance of the proposed algorithm by running several simulations and comparing successfully the results with other existing algorithms.

One of the main drawbacks in underwater communications is the low available data rate due to the use of low frequencies. Lloret *et al.* propose the use of EM waves in the 2.4 GHz ISM frequency band in underwater environments when more bandwidth is needed, despite the high attenuation of the water at these frequencies. In [16], they provide an underwater wireless sensor communication performance test using devices compatible with the IEEE 802.11 standard at 2.4 GHz, in fresh water. These tests were performed in a swimming pool by using echo request and echo reply packets in an underwater point-to-point link between two sensor nodes developed by them. They provided the maximum distance between sensors, the number of lost packets and the average round trip time at different frequencies, modulations and data transfer rates. They observed that their underwater communication system had an optimum behavior at 16 cm, working at frequency of 2,432 GHz, with the BPSK and QPSK modulations. These modulations had also good performance at distances of 17 cm, working at 2.422 and 2.427 GHz, with a percentage of lost packets slightly above 30%. They also compared their proposal with the existing systems in order to show that although it can be only used for short distances, it provides high data transfer rates. This system was proposed for precision monitoring, such as ecosystems contaminated by invasive plants, hazardous waste, and for underwater communications at deep sea, such as the neutrino telescope where they are working on.

Gao *et al.* investigate the tradeoff between the energy efficiency and the network connectivity through the selection of the transmission range in [17]. They provide an analytical framework to model the relationships between the transmission range, the average energy consumption and the connectivity in an Underwater Wireless Sensor Network scenario. They state that the selection of the transmission range needs to consider the tradeoff between the energy efficiency and the connectivity. Meeting a certain level of network connectivity incurs either cost for additional node deployment or energy due to operational deviation from the optimal transmission range. Their analytical framework provides a design guideline for energy-efficient packet transmission operation given a certain network connectivity requirement for network designers to plan the deployment of an Underwater Wireless Sensor Network.

## 4. Underwater Sensor Networks

In [18], Gómez at al. present a simple and energy-efficient localization strategy for near surface buoy-based sensors that let them self-calibrate in terms of geographic location without the need for costly and energy consuming GPS-hardware. The approach does not rely on stationary or mobile reference points. It uses the sun as a reference point. Nodes use an accurate clock and light sensors that can regularly sample the cyclic variations of light intensity resulting from the movement of the celestial bodies throughout a day. The light intensity measurements are fitted into a celestial model of the earth motion around the sun. By identifying the trajectory of the sun across the skies, they can accurately determine sunrise and sunset times, and thus extract the longitude and latitude of the sensor. The total accuracy mainly depends on the precision when identifying sunrises and sunsets. Compared to current global localization methods, the proposed system cannot be applied to tasks requiring a high accuracy, such as search and rescue, navigation, *etc.* but it can be sufficient for certain data gathering applications such as water monitoring and climate change monitoring.

In recent years, the localization problem in Underwater Wireless Sensor Networks has resulted in many innovative solutions and ideas. Han *et al.* present in [19] a comprehensive survey of localization algorithms for Underwater Wireless Sensor Networks. The classified localization algorithms into three categories based on sensor nodes' mobility: stationary localization algorithms, mobile localization algorithms and hybrid localization algorithms. Then, they compare the localization algorithms in detail and analyze future research directions of localization algorithms in Underwater Wireless Sensor Networks. They believe that the possible hot research topics are localization algorithms for large scale Underwater Wireless Sensor Networks and realistic models of sensor node's mobility according to the corresponding underwater information (e.g., depth, current velocity, water salinity, *etc.*).

Moradi *et al.* propose a novel 3D centralized localization scheme for mobile underwater wireless sensor network, named Reverse Localization Scheme (RLS) in [20]. RLS is an event-driven localization method triggered by detector sensors for launching localization process that can achieve high average response time in underwater sensor networks. It is a fast and energy efficient localization method to transfer the location estimation from the sensors to the base station. Mobile sensor nodes report the event toward the surface anchors as soon as they detect it. They do not wait to receive location information from anchors. The algorithm requires very low computational complexity and energy consumption. The authors present the design and the development of the message exchange mechanism. The proposed scheme minimizes the number of localization messages while decreases the average response time and keeps the accuracy considering to the mobility feature of water currents. It is suitable for surveillance applications that require very fast reactions to events and could report the location of the occurrence. Simulation results shows that the proposed localization algorithm improves the energy efficiency, involves low message exchange and reduces significantly localization response time with a proper level of accuracy in terms of mobility model of water currents, which can be a vital component in some applications of underwater networks like disaster prevention.

In [21], Lee and Kim describee a new range-free Localization with a Mobile Beacon for Underwater Acoustic Sensor Networks called LoMoB. In their proposal, the mobile beacon periodically broadcasts a beacon message in the underwater environment containing its location. Sensor nodes are individually localized by passively receiving the beacon messages without inter-node communications. In order to estimate the location, the system obtains a set of potential locations as candidates for a node's location using the bilateration method and then localizes the sensor node through the weighted mean of all the potential locations with the weights computed based on residuals. It improves the localization accuracy and is more tolerant to errors in the estimation of the distance between projected beacon points and sensor nodes. Simulation results show that LoMoB significantly improves the localization accuracy of other systems, especially in underwater environments that cause irregularities in the radius of the circle formed by the transceiver's beam and the location error of a mobile beacon. Simulations demonstrated that their proposal is more robust in terms of errors in distance estimation than others.

There are different sensor network architecture designs that can be used for monitoring underwater pipeline infrastructures. They are underwater wired sensor networks, underwater acoustic wireless sensor networks, Radio Frequency (RF) wireless sensor networks, integrated wired/acoustic wireless sensor networks, and integrated wired/RF wireless sensor networks. In, [22], Mohamed *et al.* discuss and compare the reliability challenges, characteristics, advantages, and disadvantages and enhancement approaches for these network architectures. The reliability evaluation has been performed in terms of network connectivity, the continuity of power supply for the network, and the physical network security. Authors developed an analytical model to compare the reliability of different architectures for monitoring underwater pipelines. Moreover, they also develop and evaluate a hierarchical sensor network framework for underwater pipeline monitoring. In this architecture, the node batteries are only used if there is a fault in the wires providing power to the nodes, so nodes would have better chance to have power supply in integrated wired/wireless sensor network than in wireless sensor networks. The integrated wired/wireless sensor network can provide good sensing and communication reliability. Moreover, they have less physical security concerns than underwater wired sensor networks because physical attacks on the network will not stop the connectivity of the network in some degree.

Macias *et al.* propose in [23] a multimedia distributed monitoring system for UWSNs, exploiting available technologies. It uses acoustic communications between underwater sensors and standard technologies, Zigbee and WiFi, for water surface communications. This integrated application-driven approach is based on a three tier architecture leveraging low cost wireless technologies. The system had a standard Charge-Coupled Device (CCD) camera for recording special underwater events, a standard signal processor and memory to store multimedia information, low cost acoustic modems, and a Zigbee or a WiFi Wireless Network Card Interface to transmit to a gateway in the surface of the water. They selected a suitable Medium Access Control (MAC) layer, T-Lohi MAC protocol, after making a comparison with some common MAC protocols. They simulated the performance of the overall system, taking into account real world parameters and models, as well as features of current standard devices, in terms of Signals Discarding Rate (SDR), signaling delay at the surface gateway as well as the percentage of true detection, pointing out good results.

In [24], Zhang *et al.* propose an architecture and protocol design for marine vehicles including Autonomous Underwater Vehicles (AUVs), Autonomous Surface Vehicles (ASVs) and Autonomous Underwater Gliders (AUGs), used as a mobile sensor network, for taking samples of a regional ocean area, ocean phenomena observing and ocean feature tracking. Authors analyze the marine vehicle dynamic control, multi vehicles mission assignment and path planning methods, ocean feature tracking and observing techniques. They integrate the control and plan of the sensor platforms with data assimilation to obtain more sample data. Combined with the observation plan in the South China Sea, authors provide numerical simulations of ocean models and data assimilation, and autonomous trajectory planning and optimization, which demonstrate the effectiveness of the system.

Climent *et al.* discuss a new architecture and routing protocol for underwater sensor networks called Energy-efficient aDaptive hiErarchical and robusT Architecture (EDETA) and EDETA-e protocol respectively in [25]. Authors have applied different delay-aware and non-delay-aware scheduling techniques, and different retransmission methods combined with them, to the architecture in order to study their performance in the underwater environment. EDETA-e is a power-aware routing protocol which organizes nodes in clusters and uses low-power modes at the times in which nodes have no need to be awake. In addition, the protocol adds fault tolerant mechanisms and has time-constrained properties. Paths can be dynamically adapted to topology changes and node failures, offering the maximum energy saving without exact location information. The authors analyzed the behavior of EDETA-e protocol in the subaquatic medium using Network Simulator 3 in terms of energy consumption, packet end-to-end delay, packet delivery ratio, number of duplicate packets, and packet loss rate. The performance evaluation of EDETA-e protocol shows a stable and efficient behavior of clusters and tree structures. Simulation results show that, taking advantage of the transmission delay when performing the scheduling can significantly reduce the energy consumption and delays, maintaining the same packet delivery ratio when packet errors are introduced. Thus, the protocol provides high reliability in terms of no data packet loss due to collisions and an optimal energy management during the normal operation phase, allowing the nodes to remain in a low-power state when they have no data to deliver to the sink. Moreover, they also proved that EDETA-e is more energy efficient than the Depth Based routing (DBR) protocol.

Taking into account that in underwater sensor networks, the probability of node failure is high because sensor nodes are deployed in harsher environments than ground-based networks, H. Min at al. propose in [26] a checkpointing scheme for the head nodes of clustering routing protocols to quickly recover from a head node failure with the aim of improving the reliability of the network. In order to achieve this goal, backup nodes that periodically save the routing information and collected data sent by head nodes. If a head node experiences a transient fault, one of the backup nodes detects the head node failure and becomes the new head node. The head node can quickly recover from a transient fault by omitting the reelection of a head node and by preventing the loss of the collected information. Even if a head node experiences a permanent fault, a backup node could become the new head node immediately. Experimental results show that the proposed scheme enhances the reliability of the networks and makes them more efficient in terms of energy consumption and recovery latency when the head node fails than other schemes without checkpointing.

In [27], Yoon *et al.* propose a new underwater routing scheme, namely Autonomous Underwater Vehicles aided Underwater Routing Protocol (AURP), which uses both heterogeneous acoustic communication channels and controlled mobility of multiple autonomous underwater vehicles (AUVs) to maximize the data delivery ratio and minimize the energy consumption of underwater sensor nodes. The network architecture consists of U-sensor nodes, gateway nodes (GWs), AUVs, and surface unit/mothership. AURP minimizes the total number of data transmissions using the following procedure. Underwater sensors send the sensed data, using a midrange underwater acoustic channel, to the GWs directly or in a multihop fashion. By exploiting controlled mobility of AUVs and the fact that AUVs can move closer to other underwater acoustic sensor nodes, a short-range high data rate underwater channel for large amount of data transmissions let GWs forward the aggregated data to an AUV. AUVs act as relay nodes, which collect sensed data from GWs and then forward them using a short-range high rate acoustic channel to the sink, which will in turn forward the data to the surface unit. AUVs have another acoustic interface for a long distance and low data rate communications to receive control signal and to send urgent data from/to the mothership via a surface unit. This procedure lets the system improve the network performance in terms of data delivery ratio and energy consumption. Authors simulated AURP performance using NS2 and they compared it with another Directed Diffusion based routing protocol. Their simulations used a realistic underwater acoustic communication channel model to evaluate the performance of the proposed scheme. Their results indicate AURP has higher delivery ratio and low energy consumption than the other. Moreover, they have observed that it could be used for applications that require mass data transmissions, such as multimedia applications.

In [28] Caiti *et al.* propose both a security suite, which is designed with the goal of reducing the communication overhead introduced by security in terms of number and size of messages between autonomous mobile underwater sensors, and a cooperative algorithm in which the mobile underwater sensors (installed on Autonomous Underwater Vehicles) respond to simple local rules based on the available information. The cooperative algorithm relies on the fact that the available information from the other sensors, though not necessarily complete, is trustworthy. It considers that when there is a loss of communication among the vehicles, the coverage performance is degraded but not lost. The proposed security suite is aimed at communication integrity and confidentiality, which jointly with the robustness of the cooperative algorithm, which is intrinsically reactive against DoS due to the emergent behaviour approach, makes it ideal to perform a mission and maintain the communication link with the network in the underwater scenario. Authors also explain the implementation details of the integration between both and provide statistics of the system performance collected from a real project. Finally, they report some results from an Underwater Acoustic Network project to characterize its application level performance including the cooperative strategy and the security suite proposed in the paper. They demonstrate that the security solution is efficient in terms of number of messages and message size, reducing the time and the energy of the transmission.

Wu *et al.* propose in [29] a series of improvements in the time-slot routing algorithm to achieve a complete routing protocol called time-slot based balancing network coding (TSBNC) routing algorithm for underwater wireless sensor networks. They designed a probability balanced mechanism and applied it to the time-slot based routing algorithm in order to increase energy consumption efficiency and extend network lifetime. Finally, they evaluated the performance of TSBNC and compared it with other classical underwater routing protocols. The simulation results show that the proposed protocol can reduce the probability of node conflicts, shorten the process of routing construction, balance energy consumption of each node and effectively prolong the network lifetime.

In [30], Navarro *et al.* examine the hydrological and biogeochemical variables obtained from the Guadalquivir estuary during three years through a real-time remote monitoring network. This network was developed with the aim of providing accurate data in order to study the influence of hydrodynamical and hydrological features within the estuary on the functioning of the pelagic ecosystem and know the trophic status of the aquatic environment. This information could be used for research or educational applications and by policy-makers or agencies in charge of the management of the coastal area. Obtained measurements showed that several sources of physical forcing (such as wind, tide-associated currents and river discharge) were responsible of the spatio-temporal patterns of the dissolved oxygen, salinity and turbidity in the estuary. The river discharge was identified as the main forcing agent of the hydrology inside the estuary. Using network values, they related episodes of elevated turbidity, together with episodes of low salinity and dissolved oxygen to the increase in water supply from a dam located upstream. The real-time monitoring network can be applied to generate early warnings for navigation, turbidity increases, hypoxia events, and toxic algal blooms, and for earlier decision making processes to prevent or mitigate undesirable events.

Balandrón *et al.* propose an Artificial Neural Network (ANN)-based architecture for completion and prediction of data retrieved by underwater sensors in [31]. The system mitigates the data incompleteness problem by employing Artificial Intelligence techniques to improve the quality of data. The system performs as follows. Magnitudes such as temperature, salinity, visibility and density are physically related one to the other, and due to proximity, they are also related to nearby values in depth and location. Under these conditions it is possible to infer and extract an approximate of the missing information from the existing data. Authors evaluate the performance of ANN-based architecture using real data to validate the approach. The proposed architecture is able to provide very low errors. Their solution is able to keep the quality of the data at near-zero cost, saving money from the costly maintenance operations. It is especially suitable for cases where there are long time failures and big portions of missing data.

## 5. Conclusions

After having organized this special issue, we can draw several conclusions from the published papers:

- Almost all the papers have highlighted the need of new technology for underwater communications because the range of applications in underwater environment is growing fast.
- Most researchers have point out the challenges and drawbacks when developing new technologies in this research field.
- The issues that should be taken into account in sensing systems are different depending on the size of the area to be monitored. It can be used one or few independent sensors to monitor a localized zone or a sensor network for large zone monitoring.
- The fact of including video cameras and the increase of the number of sensors placed inside the water has emphasized the need of higher bandwidth to transmit more data in the wireless channels.
- This Special Issue has received more papers focused about underwater architectures and protocols than the rest of the topics proposed in the call for papers.
- Although underwater sensor nodes and underwater sensor networks have been a hot topic since several years ago, it seems that this area will continue to be a hot topic for many more years to come due to the need for more technology in this field.

## Figures and Tables

**Table 1.** Classification of topics included in the Special Issue.

**Topic**	**Suggested Subtopics**
Sensor nodes	Sensor nodes to measure water parameters (salinity, conductivity, turbidity, pH, oxygen, temperature, depth, *etc.*) Sediments and pollution sensor nodes Acoustic sensors
Communications	Modems for underwater sensor networks Optical, acoustic and electromagnetic communications for underwater sensor networks
Sensor networks	Underwater sensor network architectures Wired and wireless protocols for underwater sensor networks Localization systems for underwater wireless sensor networks Target tracking using underwater sensor networks Study cases, implementations and real deployments of underwater sensor networks Routing protocols specialized for underwater wireless sensor networks Cooperation in underwater sensor networks Modeling and simulation in underwater sensor networks Data collection, storage, and retrieval by underwater sensor network

## References

[1] Garcia M., Sendra S., Lloret G., Lloret J. (2011). Monitoring and Control Sensor System for Fish Feeding in Marine Fish Farms. IET Commun..

[2] Lloret J., Sendra S., Garcia M., Lloret G. Group-based Underwater Wireless Sensor Network for Marine Fish Farms.

[3] Garcia M., Sendra S., Atenas M., Lloret J. (2011). Underwater Wireless *Ad-Hoc* Networks: A Survey. Mobile Ad Hoc Networks: Current Status and Future Trends.

[4] Martinez J.J., Myers J.R., Carlson T.J., Deng Z.D., Rohrer J.S., Caviggia K.A., Woodley C.M., Weiland M.A. (2011). Design and Implementation of an Underwater Sound Recording Device. Sensors.

[5] Ardid M., Martínez-Mora J.A., Bou-Cabo M., Larosa G., Adrián-Martínez S., Llorens C.D. (2012). Acoustic Transmitters for Underwater Neutrino Telescopes. Sensors.

[6] Baronti F., Fantechi G., Roncella R., Saletti R. (2012). Wireless Sensor Node for Surface Seawater Density Measurements. Sensors.

[7] Mànuel A., Roset X., Rio J.D., Toma D.M., Carreras N., Panahi S.S., Garcia-Benadí A., Owen T., Cadena J. (2012). Ocean Bottom Seismometer: Design and Test of a Measurement System for Marine Seismology. Sensors.

[8] Jollymore A., Johnson M.S., Hawthorne I. (2012). Submersible UV-Vis Spectroscopy for Quantifying Streamwater Organic Carbon Dynamics: Implementation and Challenges before and after Forest Harvest in a Headwater Stream. Sensors.

[9] Won T.-H., Park S.-J. (2012). Design and Implementation of an Omni-Directional Underwater Acoustic Micro-Modem Based on a Low-Power Micro-Controller Unit. Sensors.

[10] Sánchez A., Blanc S., Yuste P., Perles A., Serrano J.J. (2012). An Ultra-Low Power and Flexible Acoustic Modem Design to Develop Energy-Efficient Underwater Sensor Networks. Sensors.

[11] Shin S.-Y., Park S.-H. (2011). A Cost Effective Block Framing Scheme for Underwater Communication. Sensors.

[12] Kim Y., Park S.-H. (2011). A Query Result Merging Scheme for Providing Energy Efficiency in Underwater Sensor Networks. Sensors.

[13] Llor J., Malumbres M.P. (2012). Underwater Wireless Sensor Networks: How Do Acoustic Propagation Models Impact the Performance of Higher-Level Protocols?. Sensors.

[14] Zhang G., Hovem J.M., Dong H. (2012). Experimental Assessment of Different Receiver Structures for Underwater Acoustic Communications over Multipath Channels. Sensors.

[15] Ramezani H., Leus G. (2012). Ranging in an Underwater Medium with Multiple Isogradient Sound Speed Profile Layers. Sensors.

[16] Lloret J., Sendra S., Ardid M., Rodrigues J.J.P.C. (2012). Underwater Wireless Sensor Communications in the 2.4 GHz ISM Frequency Band. Sensors.

[17] Gao M., Foh C.H., Cai J. (2012). On the Selection of Transmission Range in Underwater Acoustic Sensor Networks. Sensors.

[18] Gómez J.V., Sandnes F.E., Fernández B. (2012). Sunlight Intensity Based Global Positioning System for Near-Surface Underwater Sensors. Sensors.

[19] Han G., Jiang J., Shu L., Xu Y., Wang F. (2012). Localization Algorithms of Underwater Wireless Sensor Networks: A Survey. Sensors.

[20] Moradi M., Rezazadeh J., Ismail A.S. (2012). A Reverse Localization Scheme for Underwater Acoustic Sensor Networks. Sensors.

[21] Lee S., Kim K. (2012). Localization with a Mobile Beacon in Underwater Acoustic Sensor Networks. Sensors.

[22] Mohamed N., Jawhar I., Al-Jaroodi J., Zhang L. (2011). Sensor Network Architectures for Monitoring Underwater Pipelines. Sensors.

[23] Macias E., Suarez A., Chiti F., Sacco A., Fantacci R. (2011). A Hierarchical Communication Architecture for Oceanic Surveillance Applications. Sensors.

[24] Zhang S., Yu J., Zhang A., Yang L., Shu Y. (2012). Marine Vehicle Sensor Network Architecture and Protocol Designs for Ocean Observation. Sensors.

[25] Climent S., Capella J.V., Meratnia N., Serrano J.J. (2012). Underwater Sensor Networks: A New Energy Efficient and Robust Architecture. Sensors.

[26] Min H., Cho Y., Heo J. (2012). Enhancing the Reliability of Head Nodes in Underwater Sensor Networks. Sensors.

[27] Yoon S., Azad A.K., Oh H., Kim S. (2012). AURP: An AUV-Aided Underwater Routing Protocol for Underwater Acoustic Sensor Networks. Sensors.

[28] Caiti A., Calabrò V., Dini G., Lo Duca A., Munafò A. (2012). Secure Cooperation of Autonomous Mobile Sensors Using an Underwater Acoustic Network. Sensors.

[29] Wu H., Chen M., Guan X. (2012). A Network Coding Based Routing Protocol for Underwater Sensor Networks. Sensors.

[30] Navarro G., Huertas I.E., Costas E., Flecha S., Díez-Minguito M., Caballero I., López-Rodas V., Prieto L., Ruiz J. (2012). Use of a Real-Time Remote Monitoring Network (RTRM) to Characterize the Guadalquivir Estuary (Spain). Sensors.

[31] Baladrón C., Aguiar J.M., Calavia L., Carro B., Sánchez-Esguevillas A., Hernández L. (2012). Performance Study of the Application of Artificial Neural Networks to the Completion and Prediction of Data Retrieved by Underwater Sensors. Sensors.

